# Encountering patients with anorexia nervosa - An emotional roller coaster. nurses’ lived experiences of encounters in psychiatric inpatient care

**DOI:** 10.1080/17482631.2022.2069651

**Published:** 2022-04-28

**Authors:** Josefine Davén, Ove Hellzen, Marie Häggström

**Affiliations:** Department of Nursing, Mid Sweden University, Sundsvall, Sweden

**Keywords:** Anorexia nervosa, encounters, lived experience, nurse-patient relationship, nursing, phenomenological hermeneutic approach, psychiatric inpatient care

## Abstract

**Purpose:**

The aim of this study was to illuminate the meaning of nurses’ lived experiences of encounters with adult patients with anorexia nervosa in psychiatric inpatient care.

**Methods:**

A qualitative phenomenological hermeneutical design was used. Personal interviews with a narrative approach were conducted with 11 nurses with experience of encountering patients with anorexia nervosa in psychiatric inpatient care.

**Results:**

Three key themes were revealed: *Being overwhelmed by emotions* consisting of three subthemes: Bearing feelings of incomprehension, Navigating emotions, and Being disappointed and frustrated; *Seeking strength to cope* consisting of three subthemes: Relying on colleagues and routines, Feeling hope and motivation, and Building inner security; and *Trying to build relations* consisting of two subthemes: Getting closer to the patient and Relating to relatives.

**Conclusion:**

Our findings illuminate the “emotional roller-coaster” which nurses are embedded in during their daily work experiences. Being able to balance one’s professional role, seeing the person behind the patient, and the illness is important in all nurse-patient encounters. An examination of nurses’ lived experiences can contribute new and important knowledge, an in-depth understanding of the nurses’ work situation, and can help identify any need for increased knowledge.

## Introduction

This study focus on care encounters with persons suffering from Anorexia nervosa, which is a debilitating, complex, and often long-term eating disorder (Brockmeyer et al., 2018; Mai et al., 2020) characterized by restricting energy intake, which leads to significantly low body weight. The eating disorder leads to disturbance of body image, an intense fear of gaining weight, negative self-image and lack of recognition of the seriousness of the illness (American Psychiatric Association, 2013; Moskowitz & Weiselberg, 2017; Seah et al., 2017). Many patients with anorexia nervosa remain ill for years, and patients are reported to have physical, psychological and social problems as a result of the eating disorder (Abbate-Daga et al., 2014; Bamford et al., 2015), which makes the recovery process slow (Mander et al., 2013; Tierney & Fox, 2009) and challenging and difficult to treat.

In Sweden, approximately 190,000 people aged 15–60 (147,000 women and 43,000 men) suffer from an eating disorder (Swedish Agency for Health Technology Assessment and Assessment of Social Services [SBU], 2019). Anorexia nervosa is one of the most serious psychiatric ilnesses with a high mortality rate (Gibson et al., 2019; Mehler & Brown, 2015). Common causes of death are suicide and complications, such as heart rhythm disorders, acute heart failure and hypoglycaemia (Arcelus et al., 2011; Seah et al., 2017; Swedish Agency for Health Technology Assessment and Assessment of Social Services [SBU], 2019). People with eating disorders are usually cared for in psychiatric outpatient care (National Institute for Health and Clinical Excellence NICE, 2017; Wentz, 2019) but in severe cases, psychiatric inpatient care is necessary. The focus is on stopping starvation and slowly increasing weight to save the patient’s life by following a predetermined structure and treatment plan based on the patient’s illness state and weight (Academy for Eating Disorders[AED], Academy for Eating Disorders [AED], 2021; Wallin et al., 2015).

Admission to the ward often is associated with anxiety and ambivalence about relinquishing control (Wallin et al., 2015). In addition to physical problems, emotional disorders, inability to be flexible, weak expressiveness, low mood and low self-esteem are common, creating barriers to communication and interaction (Moskowitz & Weiselberg, 2017; Oldershaw et al., 2019; Peterson & Fuller, 2019). The encounter between nurse and patient is central in all psychiatric care (H.E. Peplau, 1988; Holopainen et al., 2014). In healthcare, the encounter is seen as synonymous with an interaction (Holopainen et al., 2019). Travelbee (1971) sees it as a special human to human relationship, while H. E. Peplau (1987), H.E. Peplau (1988) sees it as an interpersonal process. Other authors focus on different types of encounters, such as caring, insensitive, or meaningful encounters (Halldorsdottir, 1996; Holopainen et al., 2019; L.K. Gustafsson et al., 2013; Nyström et al., 2003). In the psychiatric context, terms such as therapeutic relationship or alliance are used (Priebe & Mccabe, 2008). In the present study, a broad definition of the phenomenon was chosen to capture as many dimensions as possible in nurses’ encounters with patients with anorexia nervosa and how they interact with them.

Since healthcare professionals are responsible for establishing, initiating and inviting patients to encounters, nurses’ experiences of the encounter are important. In the literature, most of the research is about children, adolescents, or young adults with anorexia nervosa and treatment in specialized units for eating disorders. In some cases, the focus has predominantly been on physical aspects, a perspective that risks reducing the patient in the encounter, seeing only the eating disorder instead of the person behind (Salzmann-Erikson & Dahlén, 2017; Sibeoni et al., 2017a, 2017b). The encounter can be seen as the core in the care that is provided. However, research indicates that health care personnel sometimes lack the ability in seeing the person behind the illness in the encounter. Instead focus is on problems like restrictive eating and low weight (S.A. Gustafsson et al., 2021). Therefore, this study is important in bringing attention to the care encounter, and the nurse-patient relationship, from the nurses’ perspective in the general psychiatric ward setting.

To our knowledge, previous research has not illuminated this perspective, therefore the aim of this study was to illuminate the meaning of nurses’ lived experiences of encountering adult patients with anorexia nervosa in general psychiatric inpatient care.

## Materials and methods

### Design

A qualitative approach has been chosen to gain a deeper understanding of individual’s lived experience of a phenomenon (Polit & Beck, 2021). Qualitative research involves studying things in their natural setting in an attempt to make sense of, or interpret, phenomena and the meanings people bring to them (Creswell & Poth, 2017). These meanings constitute individuals’ lived experiences and can be expressed through reflection on actions in narratives (Lindseth & Norberg, 2004).

The chosen method is phenomenological hermeneutics according to Lindseth and Norberg (2004), a method suitable for making the meaning of the phenomenon between nurse and patient visible. For the meaning of people’s lived experience of a phenomenon to be available to others, the experience of the phenomenon must be narrated and transformed into text that is then interpreted (Lindseth & Norberg, 2004). According to Henricson (2017), this is made possible theoretically by using a research approach that connects phenomenology’s lifeworld theory with hermeneutics’ focus on interpretation and understanding.

### The research setting

The study was conducted in Mid-Sweden at a hospital that provides inpatient care for people over the age of 18 with eating disorders. All participants worked at a general psychiatry ward where patients with different types of psychiatric and affective disorders, such as depression, bipolar disorders, personality disorders, anxiety disorders and eating disorders, were treated. The ward had a total of 16 beds, but for patients with an eating disorder, the capacity was only one person at a time. Approximately 10 patients diagnosed with anorexia nervosa were admitted to the ward each year, several of whom were admitted several times during the period. The ward follows guidelines for investigation and treatment of eating disorders (Academy for Eating Disorders [AED], 2021). Indications for hospitalization for adult patients are severe starvation and weight loss, such as a BMI around or below 14. Some of the patients cared for at the ward had a BMI of 9–10, most of them were cared for voluntarily, but a few of them were cared for due to compulsory care because of the severeness of the illness. The most important first step in treatment is stopping starvation and starting normal eating. There is no absolute BMI value to determine how long patients will receive care, but as long as a patient has a BMI below 17, they are treated in the ward with a focus on gaining weight. At a BMI of over 17, patients can usually be transferred to outpatient care and new staff. For patients who are already known in outpatient care, contact with outpatient staff ceases during the inpatient care period.

### Participants and data collection

Narrative interviews were conducted with 11 participants, based on a model of sample size in qualitative selection and information power (Malterud et al., 2016). Malterud et al. (2016) find that it is not sample size that ensures high information power. Instead, it is the participants’ specific experiences and knowledge consistent with the aim of the study, the theoretical framework, a strong dialogue achieved through appropriate interview questions and in-depth narrative interviews. A purposive sample (Polit & Beck, 2021) was recruited, all with experience of patients with anorexia nervosa in general psychiatric inpatient care.

Inclusion criteria to participate in the study was that participants had worked at the ward at a minimum of two years. A unit manager acted as gatekeeper and forwarded the information to the entire staff (a number of 26 people) at the ward at a staff meeting, after which those interested in participating in the study contacted the first author or the unit manager, and the time and date of the interview were set.

Participants consisted of eight women and three men (median (Md) age = 42, age range = 20–67) which had worked in general psychiatric care between four and 36 years (Md = 12,5 years). Both nurses and assistant nurses was included in the study. There were five registered nurses (RNs), four of whom were specialist nurses in psychiatric care, and six assistant nurses, all with special training in psychiatric care. In Sweden, RNs have a bachelor’s degree, while “assistant nurses” study nursing care at a high school level but do not have a university degree like RNs. The RNs and assistant nurses work closely together in the team in providing nursing and take part in caring for the patient, and the everyday encounters.

Some of the participants worked in a smaller team within the ward, with a special focus on eating disorders, but all of the participants had either been a part of the smaller team before or had extensive experience with patients with eating disorders at the ward. In the presentation of the results, all participants are referred to as “nurse” to conceal their identities. Data collection was conducted through recorded, individual and narrative interviews with open-ended questions (Brinkmann & Kvale, 2015; Mishler, 1986). Due to the pandemic, the interviews were conducted by telephone, in a time chosen by the participant, and performed by the first author from May 2020 to October 2020. Participants were asked to narrate their lived experiences of encountering patients with anorexia nervosa in psychiatric inpatient care. The interviews lasted from 36 to 80 minutes (Md = 53 min). The interviews started with an opening question where participants were asked to talk freely about their experiences of patients with anorexia nervosa. The main questions included, “Tell me about your experience with people with anorexia?”, “Tell me about an encounter with a patient that evoked negative feelings?” and “Tell me about an encounter with a patient that evoked positive feelings?”. Follow-up questions, such as “Can you give an example?”, “Can you tell me more about that?” and “How did you feel?”, were asked when clarification was needed. The first author transcribed the interviews verbatim.

### Phenomenological-hermeneutic approach

The interview text was processed and analysed using a phenomenological-hermeneutic approach (Lindseth & Norberg, 2004). The process of interpreting the text went through three phases: naïve understanding, structural analysis and comprehensive understanding.

During the first phase, naïve understanding, the whole text was read through to obtain a comprehensive picture of the content. A preliminary interpretation was made in order to obtain and develop ideas about the next stage of the analysis. In the second phase, structural analysis, the perspective gained from the naïve understanding was used in order to reach the meaning of nurses’ experiences. Firstly, the text was coded into meaning units and each meaning unit was condensed to a shorter form. Secondly, transformed units were reflected on and organized into subthemes. Thirdly, themes arising from the subthemes were crystalized. In the final phase of the analysis, comprehensive understanding, an interpretation of the entire text was made with regard to the authors’ pre-understanding, naïve understanding, and structural analysis. This phase clarified the meaning of the expression for the participants’ life world (Ricoeur, 1976). Findings were reflected within the context of a theoretical framework to arrive at a deeper understanding of the text (Lindseth & Norberg, 2004). The comprehensive understanding is presented in the discussion, and the reflection can be seen as a fourth step in the analysis, which is discussed in relation to previous research.

### Ethics

This study was conducted in accordance with the Declaration of Helsinki (World Medical Association, 2013). Ethical approval was obtained from the Ethical Review Agency of Sweden (no.2020–01090). All participants in the study received information about the research orally and in writing. Participation in the study was voluntary and based on informed consent, and guaranteed confidentiality. All participants could, at any time, cease participation in the study. Contact information for the first author and supervisors was given to all participants.

## Results

The articulated fundamental meaning is presented in the present tense as it describes how the phenomenon is, i.e., utterance meaning (the meaning of the text) and not utterer’s meaning (the meaning of the participant) about the phenomenon sought (cf., Ricoeur, 1976). The result is presented in two parts, the naïve understanding, and the structural analyses.

### Naïve understanding

Encountering patients with severe eating disorders, such as anorexia nervosa, evokes an emotional response in nurses. The encounter is seen as demanding and sometimes emotional, causing nurses to re-evaluate their previous perceptions of the illness. Working according to a predetermined structure and a treatment programme helps nurses cope, as they have a protocol to rely on, especially when encountering the patient for the very first time or in situations where patients are ambivalent or resistant to receiving treatment. Nurses sometimes have encounters with relatives, which can be challenging and difficult due to the relative’s own knowledge of the illness and engagement in the treatment. This also depends on how much the patient wants their relatives to be involved. Patients who improve through treatment, become healthier and feel better, can evoke feelings of hope and joy among nurses, which contributes to a feeling of doing a good job. Encountering patients entails an emotional commitment to another person that challenges the nurse to look beyond the illness and see the person behind it.

### Structural analyses

Multiple structural analyses resulted in three themes and eight subthemes illuminating the meanings of the nurses’ lived experiences of encountering patients with anorexia nervosa. An overview of themes and subthemes is given in Table I.

**Table I.** Overview of themes and subthemes

Themes	Subthemes
Being overwhelmed by emotions	Bearing feelings of incomprehension Navigating through emotions Being disappointed and frustrated
Seeking strength to cope	Relying on colleagues and routines Feeling hope and motivation Building inner security
Trying to build relations	Getting closer to the patient Relating to relatives

*Theme I—Being overwhelmed by emotions*: This theme reflects the meaning of the very first encounter with a patient with anorexia, the difficulties understanding the eating disorder and the frustration it causes the nurse. Furthermore, this theme also captures how nurses became emotionally affected by the patient, mostly resulting in stress when encountering a seriously ill patient. The theme *Being overwhelmed by emotions* consists of the subthemes: *Bearing feelings of incomprehension, Navigating through emotions*, and *Being disappointed and frustrated*. The participants express that the impact the illness has on the patient is incomprehensible. Nurses’ expressions often revealed feelings of confusion in trying to understand the illness. The participants express feelings of sadness, despair and frustration at not being able to help or when the patient relapses and is re-admitted to the ward. The encounter also evokes feelings of compassion and pity.

*Bearing feelings of incomprehension* illuminates the difficulties related to encountering a patient with anorexia nervosa, especially when encountering a patient for the first time, but also in the nurses’ continuing work with patients. It is difficult to comprehend the severity of the illness, which reaches beyond what is seen as possible to understand. These feelings typically emerge when the nurse encounters a patient for the first time and struggles to understand the patient’s self-image and the impact of the illness. Nurses find it difficult to grasp the patient’s self-perception as overweight when the patient is so emaciated that the bones under the skin are visible:

You feel like you cannot understand this, how sick they get, in their thoughts. It’s hard to grasp. You want to be slim and starve yourself, that is one thing, but their thoughts make it so difficult that they get a twisted self-image … it’s hard to understand how they can look at themselves and say they’re fat even though they’re so skinny. It is difficult to accept that they experience it that way.

Impressions of the body raise questions, such as “how can you live and look like that? How can it go so far?”. Encounters with the severely ill patient mean feelings of own discomfort and are often emotionally shocking.

Well, it was very tough because she was so incredibly thin … honestly, you were almost nauseous when you saw her, so young and yet looked so old … there is nothing on the body … no fat, so it was just skin and bones. Every single vertebra stood out, and it was like pressure sores here and there … and well, then so the hair began to disappear. So, it’s hard to see when it’s like someone gets so consumed by illness.

Encountering severely emaciated patients with extremely low weight means feelings of puzzlement and incomprehensibility.

*Navigating through emotions* means harbouring contrasting and sometimes conflicting feelings towards the patient. The feelings are perceived as distressful and at times like an emotional roller coaster, putting aside one’s own feelings to maintain professionalism for the sake of the patient. Encountering patients in a severe state evoke feelings of deep sympathy and a will to understand and help. But at the same time, it is frightening and exhausting which evokes feelings of sadness and despair.

A lot of thoughts and feelings come and go. Trying to keep a professional role, but it does not pass unnoticed … on a personal level, you get scared like, it’s a serious condition. So, you become like, you feel for these patients and you want to help and support, but it is difficult. It is important to maintain the professional façade … in order to be able to handle this patient group.

The coexisting feelings become a challenge and means compel nurses to act professionally when dealing with their own mixed emotions in the encounter.

*Being disappointed and frustrated* means dealing with setbacks and own frustration when encountering a patient with severe anorexia, which takes a lot of energy from the nurses. It is expressed as a difficult group of patients to encounter, and involves dealing with feelings of disappointment and powerlessness when the patient does not improve or returns to previous patterns of the illness. Sometimes the patient is ambivalent or unable to receive help, due to the seriousness of the illness, and repeats the same negative pattern after the period of care. The inability to do more for the patient means feelings of defeat. The participants also report that patients sometimes try to hide food, for example, in clothing, while the nurse is sitting beside the patient during mealtime or may exercise in secret while at the ward. These situations mean feelings of disappointment and frustration among the participants.

Encountering a patient who repeatedly returns evokes questions as well as feelings of sadness and frustration regarding what happens when the patient leaves the ward. One nurse says:

… when they return, and I am thinking of one patient in particular who is admitted to the ward quite often. I mean, why don’t you get out of it, what is the reason for it, what can we do? We try everything we can in here for it to be as good as possible, and it is quite good when they go home, but then something happens …

Although they do everything they can to support and help, patients sometimes express that nurses are not good enough or committed enough. One patient even expressed this disappointment to the newspaper, which was perceived as offensive by the nurses and negatively affected them:

Well, then I got angry somehow. Because she was very demanding, we had to keep her under strict supervision so that she would not exercise … well, she did everything she possibly could to lose weight … So, we struggled with all that, and then in her eyes, we are completely useless anyway …

Facing such opinions means dealing with feelings of unfairness, disappointment and anger in the encounter.

*Theme II—Seeking strength to cope*: This theme reflects how nurses, in the encounter with the patient, experience satisfaction due to belonging to a team that works according to a predetermined structure and follows the routines of a treatment programme, and also strength in being able to talk to colleagues and support each other within the team. In addition to providing good practical care, satisfaction also affects the nurse’s self-esteem and provides a sense of security, both as a fellow human being and as a professional nurse. Working with patients with anorexia in psychiatric inpatient care means that even though it is difficult to understand and they sometimes find themselves in situations that affect them negatively, the participants feel that through experience, they gain an increased understanding of the illness. This, together with the experience of seeing other (sometimes former) patients who recovered from anorexia, means that nurses feel less anxious in the encounter with the patient. The theme *Seeking strength to cope* consists of the subthemes *Relying on colleagues and routines, Feeling hope and motivation* and *Building inner security*.

*Relying on colleagues and routines*. The importance of colleagues and belonging to a team is contributing to feelings of safety and security. When nurses feel hopelessness or meaninglessness the comradery between colleagues helps. The guidance and dialogue offered by colleagues are seen as a strength and a prerequisite for coping with tragic and difficult situations or when encountering a patient perceived as difficult. One nurse says:

I mean it’s … it happens that people, patients die. It can be someone you got to know well, who has been here often. That can be a bit heavy, but we usually talk to each other, we are quite open like that …

Also, routines and the treatment programme guides the nurses and helps maintain realistic expectations in care encounters.

Sometimes, when a nurse has to handle complex situations, or when the patient has difficulties receiving help, it is hard for nurses to be patient and there is a will to “do” rather than just “be”. Participants express that they have to remain calm in those situations, in order for changes and progress to take place. However, it can be challenging to stay hopeful and be by the patients side. A patient who has been ill for many years may never be completely free of anorexia. As the participants express it, helping a patient is a slow process, which means it is important to follow the predetermined plan.

*Feeling hope and motivation* means encounters that make the nurses feel like they are doing a good job and that their effort has meaning. These feelings arise when the patient follows the treatment programme and their mental and physical health improves, so the they can be discharged from the ward. A patient’s recovery is gratifying and provides the nurses with renewed strength and motivation in their work. They are able to share their positive experiences and carry a sense of hope to other patients they encounter. Encountering a former patient who has recovered provides satisfaction and means a feeling of a job well done, increasing motivation and commitment to their work:

… I can share with other patients who feel a strong sense of hopelessness that, (…) he or she had started to give up hope, but finally found their own thing as well, and feels good about it. And for me, of course, it evokes a lot of joy and also a hope that it is possible everything will go well.

Encounters with patients who successfully recover are a strong motivator. Hopefulness in encounters is expressed when the patient for example, no longer avoiding food or exercising excessively. When the patient is positive to receive help, nurses feel motivation and hope, positive emotions that renewed energy in their work:

When it has gone well and then you’ve met them … It is perhaps the most fun thing, that someone has come so far and had a family, for example, and had children … A person who has been very ill, who I met when she had a BMI of 9 −10 and was one of the most advanced we had, and I think that is the most fun then … there is hope in some way …

Former patients who continue to do well inspire and encourage the nurses in encounters with new patients. It evokes feelings of comfort, knowing that former patients are well and living a good life.

*Building inner security* means that over time, participants feel confident and comfortable encountering the patient and gain more knowledge about the illness and the patient’s experience. The nurses build over time their own inner security and an ability to handle different situations. As one nurse reports:

… because having met and seen many, I know that it … it is not impossible. Then there is probably a feeling of safety and security in me anyway that you like … you work for something that … it can get better. You have to endure, it’s about enduring and, and finding that acceptance as well …

When a nurse can handle complex meal situations and patients’ anxiety, the knowledge and experience provide the strength and inner security to cope with encounters with future patients.

*Theme III—Trying to build relations*: This theme reflects on encounters when, the nurse getting closer and starting to know the patient and the patient´s needs. This includes situations where they need to adapt and relate to relatives in different ways. The theme *Trying to build relations* consists of two subthemes: *Getting closer to the patient* and *Relating to relatives.*

*Getting closer to the patient* means that the nurse, due to a long period of care or repeated visits to the ward, gains deeper knowledge about the patient’s problems and needs, and adapt to the situation and the individual. During the care period, the nurse-patient relationship evolves and the patient gains confidence in the nurse. Having good knowledge of the patient also means being affected by the patient’s condition and emotions. When the patient is in poor condition and the nurse-patient relationship is new, the patient’s extreme anxiety can cause stress and it can be hard to bear. After getting to know the patient better, nurses become more comfortable in the encounter:

… the most important thing is seeing them and talking, because it will be a long time, it will be a struggle … So, that’s probably the most important thing, that they get a good encounter, so that they endure and so that they can continue the fight.

The participants told that a bond between nurse and patient is usually created over time, and the nurse becomes more and more invested in the patient. This facilitates the relationship and the interaction, meaning that the nurse can provide better support and the patient is more likely to open up and trust the nurse.

*Relating to relatives* means that nurses’ encounters with relatives are important, complex and sometimes difficult, as relatives are concerned for their loved ones and even sometimes have their own problems with food and eating. The nurses told that they often are unsure about the relatives’ views on anorexia and their insight into the eating disorder, and treatment in the ward. Furthermore, participants told that they sometimes notice that family members are also unwell and severely affected by the patient’s condition, or do not realize the severity, and lack insight into the illness.

The nurses can also feel worry and concern about the relatives, it is hard to see them suffering while trying to deal with the situation and remain supportive to the patient, their child or partner. Encountering relatives, parents, or boyfriends, for example, also means that nurses feel anxious about their well-being and how long they will cope and support the patient:

You get affected; you cannot avoid it. And then to meet relatives who also are in crisis and have difficulties dealing with it, but you have to have a professional role in the treatment. Although it also affects you emotionally.

The participants expressed that even though patients are over 18 and are seen as adults, many of them live at home with their parents. The parents often have strong opinions about treatment and how things should be done, but the patient is the one who decides over their own treatment, which creates a complicated situation for the nurse. It means that the nurse often has to explain and defend the arrangement for the patient’s treatment and inform parents about the planning for the patient’s stay in the ward.

As mentioned, the patient is often ambivalent to receive treatment or does not understand the importance of treatment due to the seriousness of anorexia. These situations are sometimes even more complicated when relatives are involved. Participants describe situations where the relatives’ presence aggravates the situation and that it can be difficult to respond and relate to the relatives’ opinions, especially when they do not have insight into the severity of the patient’s condition, enable the patient’s behaviour and attempt to advocate for the patient.

There was a patient in here, she was very ill and had a BMI of 11 or something like that … she had her mother on her side, convinced that it was not an eating disorder … that fight with a parent, you do not want that. Because she wanted to be involved in controlling and supporting her child, that it was not the eating disorder that was the problem, that’s not why she lost weight and all that.

These encounters mean a struggle for nurses, who have to meet the relatives in a respectful way while trying to help the patient recover.

## Comprehensive understanding and Discussion

The aim of this study was to illuminate the meaning of nurses’ lived experience of encountering adult patients with anorexia nervosa in psychiatric inpatient care. We found three themes that illuminate interviewees’ lived experiences: “Being overwhelmed by emotions”, “Seeking strength to cope”, and “Trying to build relations”. Patient´s expressions emotionally affected nurses’ caring actions as well as their self-esteem and moral identity in the encounter. It is apparent that encountering patients with anorexia pervades the nurses’ work and affects them deeply as a person. Contradictory feelings towards individuals with anorexia and an openness to the patient’s suffering and vulnerability touch the nurse’s own vulnerability, leading to a protective and caring approach where the nurse cares about and for the patient (cf., Christensen & Hewitt-Taylor, 2006; Martinsen, 2018).

In the present study, nurses are challenged when being overwhelmed by emotions, which indicates that encountering patients with anorexia must be seen from an ethical and holistic perspective, which is also supported in the literature (e.g., Bezance & Holliday, 2013; Medway & Rhodes, 2016). However, being emotionally affected by the patient is not only an obstacle, it is also a strength and a prerequisite for good clinical care (Andreassen Devik et al., 2013; Martinsen, 2018). Clinical nursing includes human encounters with vulnerable people, power relations and professionalism expressed in a societal and cultural context (Martinsen, 2021) where the nurse is influenced and emotionally affected (Pemberton & Fox, 2013). It can be a difficult balancing act for a nurse to take responsibility for the care of a patient while encouraging the patient to take greater responsibility for their own recovery (Van Ommen et al., 2009).

According to S.A. Gustafsson et al. (2021) the focus in inpatient care is on physical recovery and normalization of eating and weight, and not primarily on the psychological distress. This may explain why nurses have difficulty developing therapeutic relationships with these patients. Another reason may be that when one is overwhelmed by conflicting emotions, as evidenced by this study, the encounter and care risk becoming paternalistic, that is, an institutional construct that oversteps a patient’s preferences, decisions or actions in support of their overall treatment, which stands in opposition to alliance building (Breier-Mackie, 2001). Working in inpatient psychiatric care with patients with anorexia challenges the nurses’ identity as healthcare professionals. Finding a balance between competing and conflicting emotions and tasks, for example, risk assessment and compliance with treatment in recovery-oriented care, autonomy and the promotion of patients’ rights, can cause moral distress. A fact that indicates that elements such as professionalism and care are closely intertwined with the nurse as a person. Nurses may experience moral distress as they deal with experiences of incomprehensibility, confusion and frustration in parallel with compassion for the patient. Kertchok (2015) describes working in pychiatric care as a challenge, facing many different situations and concerns, and nurses combine knowledge, experience and skill to care for patients. But when a nurse has feelings of failure in the encounter with the patient or in performing the job, the question arises: Have I done the best I can? (Musto & Schreiber, 2012). However, this study and others (e.g Burston & Tuckett, 2012; Kertchok, 2015; Musto & Schreiber, 2012) show that conversations with other colleagues can help nurses cope and reduce feelings of moral distress.

The present study shows that working in a team according to a predetermined structure, routines and a treatment programme can be strengthening (cf., Karlsson et al., 2020). This affected their self-esteem as they were seeking strength to cope and gave them a sense of security, both as fellow human beings and as a professional. Having peers to rely on and feeling a sense of comradery in the team was positive, but can also be a risk if the team views the patient negatively because of experiences of hopelessness and frustration that emerge (cf., Seah et al., 2017). This could affect the interaction between patient and nurse and may lead nurses to treat patients differently based on their own positive or negative experiences in patient encounters (cf., Hellzén & Asplund, 2006). However, the team also acts as an oasis, where nurses support each other and show understanding while gaining new strength to encounter severely ill patients. Hiding behind professionalism or using it to shield oneself from one’s own emotions may be a risk and can be perceived as ignorance, disengagement and insecurity on the part of nurses, which Lewin et al. (2001) mention as shortcomings in encounters between nurses and patients. Another risk reported in patient encounters was that nurses experience negative feelings towards patients who frequently needed inpatient care. According to Wise-Harris et al. (2017), Cleary et al. (2012), Buus (2011), and Koekkoek et al. (2006), patients were described as difficult, hard to treat and not susceptible to psychiatric interventions. On the other hand, our study also showed that when nurses experienced encounters with several patients over time, they gained knowledge and felt more confident and comfortable in the encounter. Nurses could act flexible and secure in their ability to master different situations and serve the patient’s needs, which also affected the patient in a positive way (cf., Fox & Diab, 2015; Gulliksen et al., 2015; Karlsson et al., 2020). Taken as a whole, this indicates that nurses working with patients with anorexia nervosa need support in their role, and recurrent education about eating disorders can help reduce nurses’ frustration in the encounter.

The present study shows that nurses got to know the patients over long periods of care or when patients returned to the ward repeatedly, and through that tried to build relations. In order to be professional and manage to build relations in encounters with patient’s, nurses have to be present and genuine. This allows nurses to get close to the patient and build a positive nurse-patient relationship, which has also been expressed by Benzein et al. (2012). This study also showed that nurses had to adapt and relate to relatives and adjust to sometimes complicated situations that arose in the encounter. Even though nurses did not always agree with the relatives’ opinions, they had to meet the relatives respectfully while supporting the patient. Though it can be a challenge, Kertchok (2015) contends that nurses have a major responsibility to support patients‘ relatives.

Every relationship holds unspoken demands, such as an ethical demand in encountering a person who needs one’s help. Ethical demands are unspoken in nature and are based on trust, love and sympathy. Encountering another human being in healthcare means holding a part of the other’s life in your hands (Løgstrup, 1994). Nurses are affected when faced with the patient’s vulnerability, and they are able to—and feel a need to—help the person in need. Feeling secure helped the nurses to be emotionally affected by the patient’s vulnerability. Even if patients’ expressions may be painful and frightening, nurses must have the courage to stay with the patient as an inter-human act where the nurse is touched by the patient’s suffering. This touches the nurse’s own vulnerability when each of the actors turns to each other in a person-to-person encounter in which they are guided by their vulnerability. According to Løgstrup (1994), vulnerability is a fundamental condition of human life, and encountering another’s expression is an interpersonal act between people guided by our perception and vulnerability.

The nurses expressed that they needed to be open to the patient’s suffering and act in a life-saving manner at the same time. Building relations was not always easy, and the encounter with the patients was perceived as challenging. One reason was that encounters are seldom voluntary, which made it difficult to establish interpersonal interactions and thoughtful meetings and risked resulting in superficial nursing (Marynowski-Traczyk & Broadbent, 2011; Waldemar et al., 2019) focused on tasks and administration at the expense of developing interpersonal interaction and person-centred relations (Wyder et al., 2017).

## Conclusion

In order to understand the situations nurses experience, it is important to consider the meaning of nurses’ lived experiences when encountering the patient. Our findings illuminate the “emotional roller-coaster” which nurses are embedded in during their daily work experiences, such as struggling to understand the complexity of illness, the need for support and education, accepting the patient as a whole person, and creating a caring relationship that corresponds to what Wright and Hacking (2012) and Ramjan (2004) describe. Being able to balance the professional role, the person behind the patient, and the illness is important in all kinds of nurse-patient encounters. By identifying the nurses’ experiences, new and important knowledge can be added to the care of people with eating disorders and contribute an in-depth understanding of nurses’ work situation while identifying any need for further knowledge about the patient group. To be able to balance all aspects in encounters with patients with anorexia, it seems of great importance that the nurses require, and are also offered, their own support to manage to cope with their role.

The results from this study may be relevant for caregivers and can be used for educational purposes and in further research in this area. This study is part of a research project focusing on adult people with eating disorders and the healthcare professionals who encounter them. Further research may focus on patients’ and relatives’ experiences of encounters in general psychiatric care to shed light on other perspectives on the phenomenon.

## Methodological considerations

The phenomenological perspective focuses on the persons lifeworld and lived experience, and thus requires openness to the interviewee’s experiences (Lindseth & Norberg, 2004). The interpretation presented in this study is the one we found to be the most probable. According to Ricoeur (1976), being able to reach and see the lifeworld goes through interpretation of the narrative, using both understanding and explanation. The first author had experience of working with patients with eating disorders but not in the same context, and had no connection to the staff or the ward. This can be seen as a strength in that sense that the participants hopefully felt free to narrate their experiences without any obligations to the first author. But it can also be seen as a limitation that the first author was not known for them and could create a barrier, not being able to see each other during the interviews. Not all the authors had prior experience working with patients with eating disorders, which was seen as a strength as it allowed the text to be viewed from a different standpoint. All the interviews were conducted by telephone due to the pandemic, which may have prevented the participants from speaking freely and feeling comfortable. However, the interviews were rich and in line with what other studies have described, participants were relaxed, willing to talk freely and being able to disclose sensitive information on the phone. Qualitative data, conducted by phone have been judged to be rich, vivid, detailed and of high quality (Novick, 2008). The first author conducted all interviews, transcribed the text verbatim and carried out the initial analysis. However, according to issues of trustworthiness, all three authors were involved in the analysis and reflection, and critically worked with the assessments until a consensus was reached and stayed true to the method. This resulted in enriching discussions that further helped the first author to curb the preunderstandings.

In the interviews, the two main questions were formulated as either positive or negative, which suggested that the participants’ narratives could be affected in either direction. By asking for both negative and positive experiences from encounters with patients with anorexia, the intention was to trigger the participants’ memory when narrating. It can be seen as a strength that the data collection was made in a ward that is not a specialist unit for eating disorders, but also cares for people with other mental illnesses. The result from this study is transferable to other contexts due to the fact that not all institutions and wards are specialized in eating disorders. This study does not present an absolute truth, but sheds light on nurses’ lived experiences of encountering patients with anorexia nervosa in general psychiatric inpatient care, and hopefully encourages more research and further reflections on how patients’ expressions impact nurses’ emotions and the care they provide.
